# Methods and efficacy of extracellular vesicles derived from mesenchymal stromal cells in animal models of disease: a preclinical systematic review protocol

**DOI:** 10.1186/s13643-019-1242-y

**Published:** 2019-12-12

**Authors:** Alvin Tieu, Mitchell Slobodian, Dean A. Fergusson, Joshua Montroy, Dylan Burger, Duncan J. Stewart, Risa Shorr, David S. Allan, Manoj M. Lalu

**Affiliations:** 1 Department of Cellular and Molecular Medicine, University of Ottawa, Ottawa, Canada; 2 Clinical Epidemiology Program, BLUEPRINT Translational Research Group, Ottawa Hospital Research Institute, Ottawa, Canada; 3 Regenerative Medicine Program, Ottawa Hospital Research Institute, Ottawa, Canada; 4 Department of Medicine, University of Ottawa, The Ottawa Hospital, Ottawa, Canada; 5 Department of Surgery, University of Ottawa, The Ottawa Hospital, Ottawa, Canada; 6 Department of Nephrology, University of Ottawa, The Ottawa Hospital, Ottawa, Canada; 7 Kidney Research Centre, Ottawa Hospital Research Institute, Ottawa, Canada; 8 Learning Services, The Ottawa Hospital, Ottawa, Canada; 9 Department of Anesthesiology and Pain Medicine, University of Ottawa, The Ottawa Hospital, Ottawa, Canada

**Keywords:** Mesenchymal stromal cells, Mesenchymal stem cells, Exosomes, Microvesicles, Extracellular vesicles, Systematic review protocol, Preclinical

## Abstract

**Background:**

Over the past decade, mesenchymal stromal cells have been increasingly investigated for their therapeutic potential in several different illnesses. However, cell therapy can be limited by potentially serious adverse events including cell embolus formation and tumorigenesis. Importantly, the protective effects of mesenchymal stromal cells are largely mediated by paracrine mechanisms including release of extracellular vesicles. This systematic review intends to synthesize the current knowledge of mesenchymal stromal cell-derived extracellular vesicles as a therapeutic option for preclinical models of disease, inflammation, or injury.

**Methods:**

A systematic literature search of MEDLINE, Embase, and BIOSIS databases will be conducted. Interventional preclinical in vivo studies using extracellular vesicles derived from any tissue source of mesenchymal stromal cells will be included. Studies will be screened by abstract, and full-text by two independent reviewers. Eligible studies will undergo data extraction with subcategorization into domains based on disease. Methods utilized for extracellular vesicle characterization and isolation will be collected, as well as information on interventional traits, such as tissue source of mesenchymal stromal cells, dosage regimen, and vesicle modifications. Reported outcomes will be collected to determine which diseases studied may be impacted most from treatment with mesenchymal stromal cell-derived extracellular vesicles.

**Discussion:**

This systematic review will summarize preclinical studies investigating the therapeutic efficacy of both small and large extracellular vesicles derived by mesenchymal stromal cells. Extracellular vesicles represent a possibility to harness the benefits of mesenchymal stromal cells with added benefits of reduced manufacturing costs and an improved safety profile. Hence, there has been an exponential increase in interest for developing this cell-free therapy with hundreds of preclinical studies published to date. However, a vast amount of heterogeneity between groups relates to methods of extracellular vesicle isolation, characterization, and study design. This review will capture this heterogeneity and identify the most commonly used and optimal approaches to evaluate mesenchymal stromal cell-derived extracellular vesicle treatment. A meta-analysis of outcomes within each disease domain will help elucidate which fields of research demonstrate promise for developing extracellular vesicles as a novel cell-free therapy. Summarizing this robust information on extracellular vesicles as an intervention can provide guidance for designing preclinical studies with hopes of future clinical translation.

## Background

Mesenchymal stromal cells represent a subset of adult stem-like cells which have been extensively investigated for therapeutic potential in a variety of disease states. Mesenchymal stromal cells have been isolated from numerous tissue sources including adipose tissue, umbilical cord, and synovial membrane [1], and they can be derived from induced pluripotent stem cells [2]. Their multilineage differentiation potential, in vitro expansive capacity, and ability for immunomodulation has led to nearly 500 clinical trials [3], including studies of septic shock [4], acute respiratory distress syndrome [5], and ischemic heart disease [6]. In particular, mesenchymal stromal cells have received approval as a therapy to treat graft versus host disease in New Zealand, Canada, and Japan [7, 8]. Cell therapies may be limited by potentially serious adverse events of cell embolus and tumor formation [9]. Thus, there is immense interest in harnessing the therapeutic effects of mesenchymal stromal cells in ways that avoid potential harms.

Mesenchymal stromal cells’ effects are largely mediated by paracrine mechanisms, and so mesenchymal stromal cell-derived extracellular vesicles may represent a safer cell-free alternative to whole cell injections. Extracellular vesicles contain biologically active factors and signaling molecules to facilitate intercellular communication. With opportunities for diminished manufacturing and storage costs for extracellular vesicles, they can also be produced as a more readily available “off-the-shelf” product, as extracellular vesicles are more stable than whole cells when cryogenically frozen. Research into the development of cell-free therapies has focused primarily on two types of extracellular vesicles, medium-large vesicles known as microvesicles, and small extracellular vesicles, which include exosomes.

**Fig. 1:**
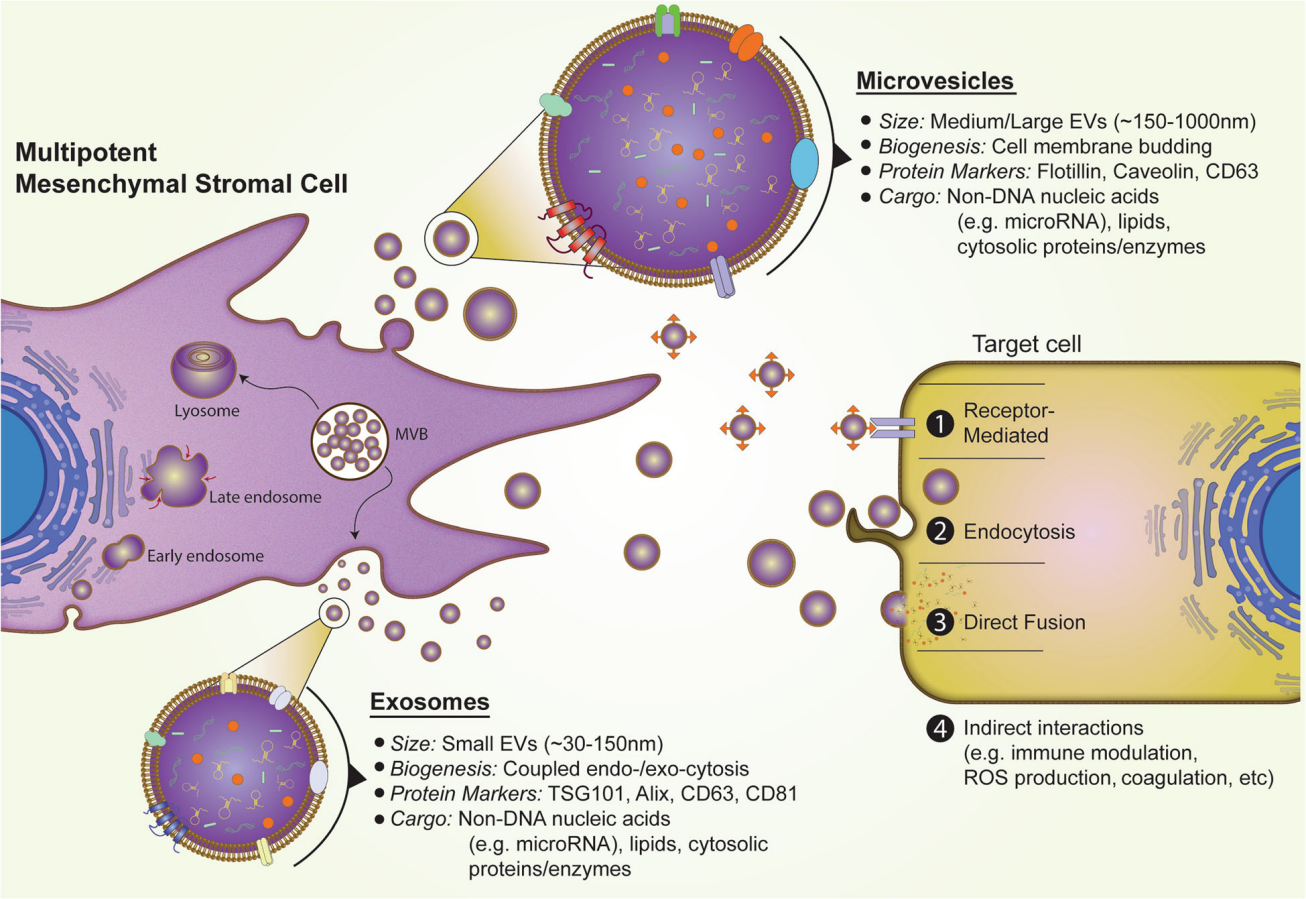
Overview of extracellular vesicles derived from mesenchymal stromal cells. Size, biogenesis, protein markers, and vesicular cargo for both exosomes and microvesicles are described. Potential mechanisms by which extracellular vesicles communicate with target cells include receptor-mediated interactions, cellular endocytosis, direct fusion with cellular membrane, and indirect interactions such as immune modulation, reactive oxygen species production, and coagulation. *MVB*, multivesicular body; *TSG101*, tumor susceptibility gene 101; *ROS*, reactive oxygen species

Microvesicles and exosomes are subsets of extracellular vesicles that range from 150–1000 and 30–150 nm in diameter, respectively [10] (Fig. 1). Both microvesicles and exosomes are prolifically produced by mesenchymal stromal cells [11] and are believed to carry bioactive cargo, such as microRNA, transcription factors, growth factors, and other regulatory proteins that help mediate their potentially efficacious properties [12]. Extracellular vesicles may serve as ideal vesicles for encapsulating therapeutic products, protect these products from degradation, and aid their delivery to specific target tissues. The opportunity to introduce or manipulate the expression of contents inside extracellular vesicles (e.g., through overexpression of protective microRNAs) has created an intriguing new domain of regenerative medicine.

The systematic review stemming from this protocol will synthesize current evidence on mesenchymal stromal cell-derived microvesicles and exosomes as therapeutic options in preclinical models of disease, inflammation, or injury. A scoping review previously published by our group systematically reviewed preclinical data on extracellular vesicles up to 2013 and found that sixteen of seventeen preclinical studies demonstrated therapeutic benefits associated with administration of mesenchymal stromal cell-derived extracellular vesicles [13]. Subsequently, the terminology surrounding extracellular vesicle research has matured, and interest in studying microvesicles and exosomes has increased exponentially, necessitating a re-evaluation of this body of evidence.

### Research objectives

We will review in vivo preclinical studies evaluating mesenchymal stromal cell-derived extracellular vesicles as an intervention for any animal model of disease or injury. Our primary objective will assess the methodology being utilized in these animal studies including (1) interventional characteristics, (2) methods of extracellular vesicle enrichment and characterization, and (3) experimental design. Given the expected heterogeneity of methods used (e.g., cell sources, isolation techniques) and outcomes assessed across various disease domains, a synthesis of these issues is needed to provide context for any subsequent meta-analysis. For our secondary objective, studies will be categorized by disease domains with subsequent meta-analyses of study outcomes to determine the therapeutic efficacy of mesenchymal stromal cell-derived extracellular vesicles in different conditions. Results of this analysis will help guide future preclinical studies of extracellular vesicles and inform whether possible human participant studies are feasible.

## Methods and design

### Review team

The team members for this systematic review encompass researchers across different scientific disciplines. The team has expertise in systematic reviews (DAF, MML, JM, DSA), basic science studies investigating novel cell therapies (AT, MML, DJS, DSA), and translational cell therapy trials (DJS, DAF) [6, 14, 15]. As well, DB participated in the development of the recently published Minimal Information for Studies of Extracellular Vesicles 2018 (MISEV2018) position statement and provides considerable experience in the field of extracellular vesicle research [16]. The student leads for this project (AT and MS) have experience in both basic science and clinical epidemiology and will be the two primary reviewers for article selection and data extraction.

### Protocol

This systematic review protocol is reported in accordance with the Preferred Reporting Items for Systematic reviews and Meta-Analysis Protocol (PRIMSA-P) reporting guidelines (Additional file 1) [17]. A summary of the protocol has been registered at the International Prospective Registry of Systematic Reviews (PROSPERO CRD42019123918).

### Eligibility criteria

#### Population

Inclusion—in vivo preclinical animal models of disease, organ injury, tumor growth, or altered immune response. Exclusion—ex vivo, in vitro, and invertebrate animal models will be excluded.

#### Intervention

Inclusion— mesenchymal stromal cell-derived extracellular vesicles including microvesicles (150–1000 nm) and exosomes (30–150 nm). Extracellular vesicles can be derived from xenogeneic, syngeneic, or allogeneic mesenchymal stromal cells from any tissue source. All routes of intervention administration will be considered, including direct tissue, intravenous, and intra-arterial injections. Experiments involving pre-treatment of mesenchymal stromal cells, co-treatment, and/or genetic manipulation (e.g., over- or under-expression of genes) before extracellular vesicle isolation will be classified as “modifications” for subgroup analysis. Exclusion—studies where extracellular vesicles are not directly administered to animals as a therapy (e.g., used as a pre-treatment for other interventions) or investigated only for their biodistribution will be excluded.

#### Comparators

Studies with any comparator (e.g., vehicle control, placebo, mesenchymal stromal cells, and fibroblast microvesicles) will be considered.

#### Outcomes

Inclusion—studies that provide information regarding intervention characteristics, methodologic approaches and experimental design parameters utilized in studies will be included. For the purposes of a potential quantitative synthesis and meta-analysis, we will extract each study’s primary outcome. If a primary outcome has not been outlined in the study, expert opinion or consensus statements will be sought to determine which in vivo outcome may be most clinically relevant for diseases treated.

#### Study design

Inclusion—interventional in vivo studies with a comparator-control will be included. Studies may be randomized, pseudo-randomized, or non-randomized. Exclusion—unpublished gray literature, abstracts, review articles, editorials, and letters will be excluded. Single arm interventional studies will also be excluded.

### Data sources

We will search Ovid MEDLINE®, Ovid MEDLINE® In-Process & Other Non-Indexed Citations, Embase Classic + Embase, and BIOSIS from inception to present day. In addition, hand searches of the bibliographies of included articles and relevant reviews will be performed.

### Search strategy

The search strategies (Additional file 2) to be used for this review will be generated by a health sciences librarian (RS) with experience designing systematic literature searches. A second information specialist with no association to the project will also review the strategy using Peer Review of Electronic Search Strategies (PRESS) before the final search procedure is executed [18, 19]. The search strategy will utilize both calculated vocabulary and MeSH terms (e.g., mesenchymal stem cells, mesenchymal stromal cells, extracellular vesicles, exosomes, and microvesicles), acronyms (e.g., MSCs, microvesicles, and extracellular vesicles), and MeSH terms with alterations as required per database. Our previous search strategy published in 2015 was updated to reflect evolving terminology in the extracellular vesicle field [13]. Preclinical filters will also be applied to improve search efficiency [20–22]. No search restrictions will be created for language or publication date.

### Study records

#### Data management

Search results will be uploaded to Distiller Systematic Review Software (DistillerSR, Evidence Partners, Ottawa, Canada). DistillerSR is an online software that allows for improved transparency, reproducibility, and accessibility for literature review.

#### Selection process

Two reviewers (AT, MS) will independently screen titles and abstracts from search results using the predefined inclusion criteria outlined above. A calibration test involving sets of 10 studies will be performed (AT, MS) to refine the screening question and ensure high inter-rater correlation (*kappa* > 0.8), prior to formally commencing the screening process. For all titles that appear to meet the inclusion criteria or where there is any uncertainty, we will obtain the full-text article. Two reviewers (AT, MS) will assess the eligibility of full-text articles. A calibration test involving a set of 10 studies will be performed (AT, MS) to ensure high inter-rater correlation. After every calibration test, the entire review team will be consulted to resolve any issues concerning full-text inclusion. After refining the full-text screening criteria, a formal screening process (AT, MS) will be commenced. Any disagreement will be resolved through discussion with a third-party member (MML, DSA). Reasons for study exclusion at this level will be recorded. There will be no restrictions on language. Articles of non-English text will be translated by native speakers at the Ottawa Methods Centre.

#### Data collection process

A standardized data extraction form will be designed by the review team and uploaded to DistillerSR. Information will be extracted independently and in duplicate from each eligible study (AT, MS). Calibration exercises of 10 studies will be conducted, and the review team will be consulted to refine the data extraction form. After commencing formal data abstraction, any disagreements will be first discussed between two independent reviewers (AT, MS). If no resolution can be made, a third-party team member will be consulted (MML).

#### Data items

Broad categories of data items include methods of vesicle separation and characterization (Table 1), intervention details (e.g., tissue type of mesenchymal stromal cell, dose, route of administration, and timing of administration) (Table 2), study characteristics (e.g., study design, population of species, model of disease) (Table 3), and preclinical outcomes.

**Table 1:** Isolating and characterizing mesenchymal stem cell-derived extracellular vesicles

Question of interest	Example answers
Terminology used	Extracellular vesicles
	Exosomes
	Microvesicles
	Small EVs
	Large EVs
	Microparticles
Size of EVs	Small EVs (30–150 nm)
	Large EVs (150–1000 nm)
	Both used together
	Both used separately
Method of EV enrichment	Ultracentrifugation
	Isolation kit
	Tangential flow filtration
Method of EV characterization	Nanoparticle tracking analysis (size)
	Dynamic light scattering (size)
	Tunable resistive pulse sensing (size)
	Western blot (protein content)
	Flow cytometry (protein content)
	ELISA (protein content)
	Proteomics (protein content)
	Electron microscopy (morphology)
	Bradford/BCA assay
Protein markers used	CD63 (seen in many EVs)
	CD9 (seen in many EVs)
	CD81 (small EV)
	TSG101 (small EV)
	Other (specify)
Negative protein markers	Calnexin
	Cytochrome C

*EV,* extracellular vesicle; *ELISA*, enzyme-linked immunosorbent assay; *BCA assay*, bicinchoninic acid assay; *TSG101*, tumor susceptibility gene 101

**Table 2:** Interventional traits and dosage regimen of the extracellular vesicle therapy

Question of interest	Example answers
Storage of EVs	Frozen at – 80 °C
	Fresh / fridge
	Not described
Tissue source of MSCs	Bone marrow
	Adipose
	Umbilical cord/Wharton’s jelly
Animal source of MSCs	Human source
	Mouse source
	Rat source
EV immunocompatibility	Xenogeneic
	Allogeneic
	Autologous
	Not described
Modification of EVs	Unmodified
	Modified MSCs before EV isolation
	Modified EVs directly
Route of administration	Intravenous
	Direct tissue injection
	Subcutaneous
	Intra-arterial
EV dosage units	EV protein amount
	EV particle number
	EV dose based on MSC cell count
Timing of First EV dose	Post-injury induction (treatment)
	Pre-injury induction (prevention)
Number of doses used	Single dose of EVs
	Multiple doses of EVs

*EV,* extracellular vesicle; *MSC*, mesenchymal stromal cells

**Table 3:** Disease domains, preclinical models, and randomization

Question of interest:	Answers
What was the disease or condition of interest?	Brain (e.g., stroke, traumatic brain injury)
	Kidney (e.g., acute kidney injury, chronic kidney disease)
	Lung (e.g., acute lung injury, pulmonary hypertension)
	Bone/joint (e.g., osteoarthritis)
	Liver (e.g., hepatotoxicity, chronic liver disease)
	Cardiac (e.g., myocardial infarction)
	Cancer
Animal model	Mouse
	Rat
	Pig
Were animals randomized?	Yes
	No

### Risk of bias assessment

For each included study, risk of bias assessments will be carried out by two independent reviewers (AT, MS) using the Systematic Review Centre for Laboratory animal Experimentation (SYRCLE) risk of bias tool [23]. The SYRCLE tool was adapted from the Cochrane Risk of Bias Tool to assess the methodologic quality using criteria specific to animal studies. Items in this tool include assessments for selection bias (sequence generation, baseline characteristics, allocation concealment), performance bias (random housing, blinding), detection bias (random and blinded outcome assessment), attrition bias (completeness of outcome data), and reporting bias. Each parameter of bias for each included study will be scored as having a low, high, or unclear risk of bias. Any disagreements between the two reviewers will be resolved through discussion with a third-party member (MML).

### Data analysis

Before a meta-analysis will be deemed appropriate to execute, the heterogeneity of included studies will be assessed. A senior author (MML, DA, DB) will review the study populations and study characteristics to judge the presence of methodologic heterogeneity. If sufficient homogeneity across studies exists, a meta-analysis will be considered appropriate to carry out. Continuous endpoints will be pooled using standardized mean differences with an inverse variance random effects method [24, 25]. Dichotomous outcomes (e.g., mortality) from each study will be pooled and described as risk ratios with 95% confidence intervals using the DerSimonian and Laird random-effects method (Comprehensive Meta-Analysis v3.1) [26]. Statistical heterogeneity of effect sizes will be assessed using the Cochrane *I*^2^ statistic test [27]. Thresholds for interpretation of *I*^2^ are as follows: 0–40% (low heterogeneity), 30–60% (moderate heterogeneity), 50–90% (substantial heterogeneity), and 75–100% (considerable heterogeneity) [27]. If there is considerable heterogeneity (75–100%), sources of heterogeneity will be explored through subgroup and sensitivity analyses.

### Subgroup analyses

Planned subgroup analyses include intervention characteristics, such as tissue source of mesenchymal stromal cells (e.g., bone marrow, umbilical cord, and adipose), size of extracellular vesicles (e.g., small extracellular vesicles from 30–150 nm, or medium/large extracellular vesicles from 150–1000 nm), method of extracellular vesicle isolation (e.g., ultracentrifugation, tangential flow filtration, and size exclusion chromatography), and methods of vesicle characterization (e.g., Western Blot, electron microscopy, and flow cytometry). Additionally, studies will be subgrouped by disease domain for preclinical outcome assessment.

### Meta-biases assessment

Evaluation for publication bias and selective reporting bias will be conducted using a funnel plot, generated by Comprehensive Meta-Analyst, and Egger’s regression test [28, 29].

### Knowledge dissemination

Understanding that there has been a markedly heightened interest to evaluate the therapeutic potential of mesenchymal stromal cell-derived extracellular vesicles, this review will first assess the feasibility of conducting a meta-analysis based on the final number of included preclinical studies. If a feasible number of articles are included, our team will summarize the methodology data and conduct a meta-analysis of outcomes. If the included number of articles is too large to feasibly perform multiple analyses in a single publication, a systematic review of the overall methodology being used for in vivo extracellular vesicle studies will be first published. Subsequently, analyses for each disease domain with more details (e.g., risk of bias and outcome measures) will be published as separate manuscripts in peer-reviewed journals. Finally, findings will be disseminated through international conferences with basic and translational scientists to help guide future preclinical and clinical study design of extracellular vesicle therapy.

### Amendments

If any amendments to this protocol are necessary, the date and specific changes to the protocol will be documented on PROSPERO with rationales as to why the alterations were required.

## Discussion

Our systematic review intends to identify and summarize preclinical studies investigating the therapeutic efficacy of both small and large extracellular vesicles derived from mesenchymal stromal cells. This work updates our previous study that captured studies through 2013 [13]. Although only 17 studies were included in the previous systematic review, the therapeutic potential for this novel cell-free therapy was evident as many disease and injury states had been investigated. Since then, many more research groups around the world have begun to study extracellular vesicles, and they are now well-recognized to carry biologically active factors that mediate the therapeutic effects of mesenchymal stromal cells. Extracellular vesicles represent a novel investigative direction within regenerative medicine.

We anticipate that this comprehensive synthesis and meta-analysis of preclinical studies assessing the beneficial or adverse effects of mesenchymal stromal cell-derived extracellular vesicles will generate valuable data to help guide future preclinical study design. More importantly, these findings can help provide considerations of future translational human participant studies.

## Supplementary information

**Additional file 1.** Preferred reporting items for systematic review and meta-analysis protocols (PRISMA-P) 2015 Checklist.

**Additional file 2.** Search strategy used for systematic literature search.

## Data Availability

Not applicable.
